
JOURNAL INFORMATION
==============================
NLM Title Abbreviation: ScientificWorldJournal
Journal ID: ScientificWorldJournal
NLM Journal ID: 101131163
Journal ID: TSWJ

The Scientific World Journal

ISSN: 2356-6140
EISSN: 1537-744X
Publisher: Wiley

ARTICLE INFORMATION
==============================
PMCID: PMC6083903
PMID: 30147562
DOI: 10.1100/tsw.2001.204
Article ID: 201086
Article version: 1
Subjects: Short Report

The Super Anti-Apoptotic Factor Bcl-xFNK: A Novel Mutant of Rat Bcl-Xl with a Gain-of-Function Phenotype

Asoh Sadamitsu *
Ohtsu Takashi
Ohta Shigeo
Department of Biochemistry and Cell Biology, Institute of Gerontology, Nippon Medical School, 1-396, Kosugi-cho, Nakahara-ku, Kawasaki-city, Kanagawa-ken 211-8533, Japan
*Sadamitsu Asoh: sada@nms.ac.jp
Publication date: 2001
Electronic publication date: 2001 Dec 8
Volume: 1
First page: 91
Last page: 91
Copyright: Copyright © 2001 Sadamitsu Asoh et al.
Copyright year: 2001
License: This is an open access article distributed under the Creative Commons Attribution License, which permits unrestricted use, distribution, and reproduction in any medium, provided the original work is properly cited.
License URL: https://creativecommons.org/licenses/by/3.0/

==============================
Miami *Nature Biotechnology* Short Reports
*TheScientificWorld* (2001) 1 (S3), 91SR
ISSN 1532-2246; DOI 10.1100/tsw.2001.204

### THE SUPER ANTI-APOPTOTIC FACTOR BCL-XFNK: A NOVEL MUTANT OF RAT BCL-XL WITH A GAIN-OF-FUNCTION PHENOTYPE

Sadamitsu Asoh*, Takashi Ohtsu, and Shigeo Ohta

Department of Biochemistry and Cell Biology, Institute of Gerontology, Nippon Medical School, 1-396, Kosugi-cho, Nakahara-ku, Kawasaki-city, Kanagawa-ken 211-8533, Japan
* sada@nms.ac.jp

**INTRODUCTION.** A powerful synthetic anti-apoptotic factor would be useful for future therapeutic applications in medicine. Analysis of the crystal structure of renatured human Bcl-$x_L$ has suggested that it forms an ion channel (1). Additionally, Bcl-$x_L$ has been shown to form an ion channel in synthetic lipid membranes (2). We have independently determined the x-ray structure of native rat Bcl-$x_L$ with a 2.5 Å resolution and found it to have nine intramolecular polar interactions to stabilize the central putative pore-forming domain ($\alpha$5- $\alpha$6 helices) (3). We hypothesized that these hydrogen bonds may disturb the anti-apoptotic effect by inhibiting the insertion of the pore-forming domain into membranes. In the present study we designed a mutant Bcl-$x_L$, named Bcl-xFNK to make the $\alpha$5- $\alpha$6 helices more mobile or flexible by disturbing the formation of three hydrogen bonds which stabilize the tip of the putative pore-forming domain.

**METHODS.** To construct Bcl-xFNK, three amino acid substitutions ($Tyr^{22}$ to Phe [F], $Gln^{26}$ to Asn [N] and $Arg^{165}$ to Lys [K]) were introduced into rat Bcl-$x_L$ by a two-step PCR mutagenesis. Chinese hamster CHO K1 cells were cultured in D-MEM/F-12 medium containing 10 % fetal bovine serum. Human Jurkat cells and IL-3 dependent murine cells, FDC-P1 and BaF/3, were cultured in RPMI 1640 medium containing 10 % FBS. For FDC-P1 and BaF/3 cells, IL-3 was added in the medium. Plasmids were introduced into Jurkat, FDC-P1 and BaF/3 cells by electroporation and into CHO cells using Superfect. Stable transfectants Jurkat cells were treated with various death-inducing stimuli. CHO cell transfectants were deprived of serum. FDC-P1 and BaF/3 transfectant cells were incubated in the absence of IL-3. Surviving cells were counted by the trypan blue exclusion or by the WST-1 assay. BIOTRAK p42/p44 MAP kinase enzyme assay system was used to measure the p42/p44 MAP kinase activity of cells.

**RESULTS.** When over expressed in Jurkat or CHO cells, Bcl-xFNK was markedly more potent than wild-type Bcl-$x_L$ in prolonging survival against anti-Fas (CH-11), staurosporine, TN-16, camptothecin, hydroxyurea, trichostatin A, hydrogen peroxide, paraquat, a calcium ionophore (A23187), heat treatment and serum deprivation. Interestingly, Bcl-xFNK allowed IL-3-dependent FDC-P1 but not BaF/3 cells to grow without IL-3. In Bcl-xFNK transfectants of FDC-P1 and Jurkat cells, the p42/p44 MAP kinase was activated by 2 to 5 times, but not in BaF/3 and CHO cells. Thus, Bcl-xFNK might acquire a new function to activate the MAP kinase in a cell-type specific manner.

**DISCUSSION.** These findings of this study suggest that the central α5-α6 pore-forming region of the anti-apoptotic factor Bcl-$x_L$ has a pivotal role in suppressing apoptosis. Bcl-xFNK would be useful for making commercially important anima cell lines robust to culture insults may more effectively prevent cell death in diseases such as ischemia than Bcl-$x_L$ and Bcl-2.

**REFERENCES.**

1. Muchmore, S.W., Sattler, M., Liang, H., Meadows, R.P., Harlan, J.E., Yoon, H.S., Nettesheim, D., Chang, B.S., Thompson, C.B., Wong, S.L., Ng, S.L., and Fesik, S.W. (1996) Nature 381, 335-341
2. Minn, A.J., Velez, P., Schendel, S.L., Liang, H., Muchmore, S.W., Fesik, S.W., Fill, M., and Thompson, C.B. (1997) Nature 385, 353-357
3. Aritomi, M., Kunishima, N., Inohara, N., Ishibashi, Y., Ohta, S., and Morikawa, K. (1997) J. Biol. Chem. 272, 27886-27892
